# Environmental effects on inter-brain coupling: a systematic review

**DOI:** 10.3389/fnhum.2025.1627457

**Published:** 2025-07-31

**Authors:** Octavia Leahy, Emily Kontaris, Natalie Gunasekara, Joy Hirsch, Ilias Tachtsidis

**Affiliations:** ^1^Optics Laboratory, Department of Medical Physics and Biomedical Engineering, University College London, London, United Kingdom; ^2^Health and Well-being Centre of Excellence, Givaudan UK Limited, Ashford, Kent, United Kingdom; ^3^Yale School of Medicine, Departments of Psychiatry, Comparative Medicine, and Neuroscience, Yale University, New Haven, CT, United States

**Keywords:** functional near-infrared spectroscopy (fNIRS), inter-brain coupling (IBC), hyperscanning, environment, social neuroscience, two-person neuroscience, EEG, neuroimaging

## Abstract

**Introduction:**

Environmental factors play a critical role in shaping social interactions, and emerging evidence suggests they may also influence inter-brain coupling (IBC). The main purpose of this paper is to systematically review how environmental variables influence IBC during hyperscanning studies of social interactions. Additionally, this article provides an overview of the experimental protocols employed and identifies both opportunities and challenges within this evolving field.

**Methods:**

Following PRISMA guidelines, we conducted a systematic literature search in the PubMed and Scopus databases to identify relevant articles. Of the 106 articles initially identified, 7 met the inclusion criteria for this review. The selected articles are original research published up to February 2025, each employing hyperscanning techniques to observe IBC in response to manipulated environmental factors. Articles were excluded based on factors such as the absence of environmental manipulation or not measuring IBC as an outcome.

**Results:**

The findings reveal that IBC is significantly influenced by environmental factors such as interpersonal distance, background noise, virtual reality, and music. These factors modulate neural synchrony in brain regions critical for social cognition.

**Conclusion:**

The limited number of studies in this area reflects both the emerging nature of this research field and the challenges associated with experimental protocols and funding. Despite these limitations, this review underscores the crucial role of environmental factors in shaping IBC during social interactions. This growing field holds great potential for guiding the design of supportive social settings and targeted interventions that promote social cohesion and mental wellbeing.

## 1 Introduction

Social interactions form the very essence of our daily lives. From family gatherings to workplace collaborations, engaging with others enables us to forge bonds that shape our identity and experiences. Positive interactions, such as cooperation, collaborative problem-solving and effective communication, foster feelings of connection, respect, and shared achievement, acting as a protective factor, promoting resilience (Lincoln, 2008). Conversely, negative interactions, such as conflict, competition and ineffective communication, can act as stressors, contributing to destructive thought patterns and mental health challenges. Social neuroscience seeks to understand these dynamics, exploring the neural processes that underline emotional and cognitive experiences.

The environment in which we exist exerts a substantial influence on our social behavior (Patterson, 2015). It is not merely a backdrop but rather an active participant, shaping our perceptions and responses, often in ways that may escape conscious awareness. Decades of behavioral research underscore the profound role that physical and sensory environmental cues, such as temperature, lighting, spatial distance, and sound, play in shaping interpersonal dynamics. Sensory experiences like warmth and proximity have been shown to evoke psychological and social states that influence trust, cooperation, and emotional resonance (Anderson and Anderson, 1984; Anderson et al., 2000; Fay and Maner, 2014; Bargh and Shalev, 2011; Huang et al., 2014; Kang et al., 2010; Williams and Bargh, 2008a,2008b). Similarly, lighting has been linked to conflict resolution, with warm, bright lighting fostering more amicable behaviors (Rot et al., 2008; Baron et al., 1992). Whether physical or virtual, the environments we inhabit are far from neutral spaces; they serve as dynamic canvases that communicate values and contribute to the quality and outcomes of social interactions. Therefore, to fully understand social behavior, we must consider not only individual actions but also the broader environmental context in which these interactions unfold. Understanding these complex relationships may enable us to create more conducive spaces for positive interactions, thereby addressing pressing societal challenges related to mental health and wellbeing.

Traditional neuroimaging refers to established methods used to visualize and measure brain activity. These techniques vary in their approach, strengths, and limitations. EEG is one such method, deploying an array of electrodes on the scalp to capture minute electrical potentials associated with neuronal firing in the brain (Li et al., 2022). EEG displays exceptional temporal resolution with sample rates often exceeding 1 kHz. However, EEG does not provide information about the location of the neural computations it measures. Despite this limitation, EEG offers frequency component information that reflects different aspects of brain function.

Functional magnetic resonance imaging (fMRI), on the other hand, relies on the different magnetic properties of oxygenated hemoglobin (HbO2) and deoxygenated hemoglobin (Hb) (Glover, 2011) to indicate the locations of neural processing. During neural activity, an increase in blood flow to specific brain areas brings a higher concentration of oxygenated blood, which fMRI detects as a change in the local magnetic field, known as the Blood Oxygen Level Dependent (BOLD) signal. This technique boasts excellent spatial resolution, making it ideal for identifying specific brain regions involved in different tasks.

In contrast, functional near infrared spectroscopy (fNIRS), relies on the different optical properties of HbO2 and Hb (Chen et al., 2020) to detect signals associated with neural processes. fNIRS employs pairs of head-mounted optodes, forming measurement channels, that emit and detect near-infrared (NIR) light. The light emitted diffuses through the scalp, skull, and brain tissue and is partially absorbed and scattered. In particular, it is differentially absorbed by HbO2 and Hb. The light reflected is captured and quantified, enabling the assessment of hemoglobin concentration. While fNIRS offers a suitable temporal resolution (approximately 10 Hz) and is relatively resistant to motion artifacts, its spatial resolution is limited to superficial cortical areas, as it only probes to a depth of approximately 1.5 cm from the scalp.

Traditional neuroimaging methods have played a pivotal role in identifying the neural underpinnings of how the environment shapes social cognition. For example, Cook et al. (2018) recorded EEG responses while individuals rated faces under varying odor conditions (pleasant, unpleasant, and no-odor), using visual analog scales. Odors were found to significantly influence the perception of facial expressions, with distinct event-related potential (ERP) components revealing stages of visual processing, face perception, and emotional evaluation. Moreover, sweat, obtained from individuals in an anxiety-inducing condition, caused greater activation in brain regions associated with processing social-emotional stimuli and regulating empathic feelings (Prehn-Kristensen et al., 2009). Together, these studies emphasize the significance of olfactory stimuli in shaping our social perceptions and behaviors. Likewise, a coordinate-based meta-analysis of neuroimaging studies on music-evoked emotions revealed widespread activation in brain structures such as the amygdala, anterior hippocampus, auditory cortex, and reward networks (Koelsch, 2020). The findings underscore the rewarding nature of music, highlighting the auditory cortex as a central emotional hub as well as emphasizing the hippocampus’s role in attachment-related emotions and social bonding. Furthermore, music intervention in children with autism spectrum disorder (ASD), elicited improvements in social communication scores and enhanced resting-state brain connectivity, particularly between auditory, subcortical and frontal-motor regions (Sharda et al., 2018). Together, these findings indicate the profound impact of music not only on emotional processing but also on the neural circuits associated with social interactions, suggesting its potential as a therapeutic tool.

Traditional brain imaging studies investigating environmental influence on social interaction, such as those mentioned above, have predominantly employed single-brain approaches, where a participant views social stimuli on a screen while their neural activity is recorded. While these methods are instrumental for identifying localized brain activations, studying the social brain in an isolated context has its limitations. Social interactions are inherently dynamic, involving a continuous exchange of information. However, single-brain approaches rely on snapshots of brain activity rather than real-time assessments and, therefore, may not capture the temporal dynamics and reciprocal neural processes occurring as we interact.

In response to the limitations of single-brain approaches, a pioneering method emerged in 2002, by joining two fMRI scanners (Montague et al., 2002). This technique, termed “hyperscanning,” allowed for the simultaneous, real-time measurement of brain activity across multiple participants, revolutionizing the field of social neuroscience (Hari and Kujala, 2009; Hari et al., 2013). Beyond the traditional focus on intra-brain analyses, hyperscanning paradigms delve into the dynamics between brains, showcasing their continuous and mutual adaptation during interactions. This introduced a novel neural correlate, inter-brain coupling (IBC), whereby the neural activities of different brains synchronize and reciprocally influence each other in a dynamic manner over the course of an interaction (Hasson and Frith, 2016; Czeszumski et al., 2020). The relationship between IBC and social interactions is an area of active research. While compelling evidence links the two, much remains to be understood about the underlying mechanisms. The Dynamic Neural Coupling Hypothesis suggests that IBC, from non-task-related signals, encompasses a range of interactive functions characterized by the rapid exchange of social information (Hasson and Frith, 2016). Elevated synchronization has been associated with several positive social phenomena such as cooperation, connectedness, moments of agreement and collaboration (Kinreich et al., 2017; Hirsch et al., 2021; Nozawa et al., 2019; Hu et al., 2017; Hoehl et al., 2021; Cui et al., 2012; Toppi et al., 2016; Szymanski et al., 2017; Cheng et al., 2019), as well as activities requiring coordination, such as button pressing and singing (Funane et al., 2011; Osaka et al., 2015). Furthermore, the degree of IBC between interacting individuals is predictive of learning outcomes across various tasks (Pan et al., 2020). Notably, this synchrony is particularly prevalent in brain regions such as the angular gyrus (AG) and occipital-temporal areas, which are vital for processing faces and social behavior (Piva et al., 2017). The specific behaviors associated with this neural phenomenon remain an open question, and it is likely that IBC represents one piece of a broader puzzle. Moreover, it is important to note that coherence between individuals can also be observed through other physiological and behavioral measures, which may complement or provide alternative insights to IBC. For example, synchrony of pupil diameter as well as heart rate has been shown to increase during joint attention (Wohltjen and Wheatley, 2021; Flory et al., 2023; Sharika et al., 2024). These physiological markers, like IBC, point to shared states between individuals that may underlie various aspects of social interaction and connection. A comprehensive understanding of social interactions, therefore, requires integrating these diverse measures to explore how coherence manifests across neural, physiological, and behavioral domains.

A diverse array of advanced analytical tools have been applied to compute IBC, contributing to variations and discrepancies across studies, as discussed by Hakim et al. (2023). There is a pressing need for increased clarity regarding the capabilities and limitations of each analytic method to ensure coherent and consistent advancement in the field. This becomes particularly pertinent when associating IBC with specific cognitive mechanisms or stating its potential as a metric for psychiatric treatment (Osaka et al., 2015; Dravida et al., 2020; Ono et al., 2022; Leong and Schilbach, 2019).

**FIGURE 1:**
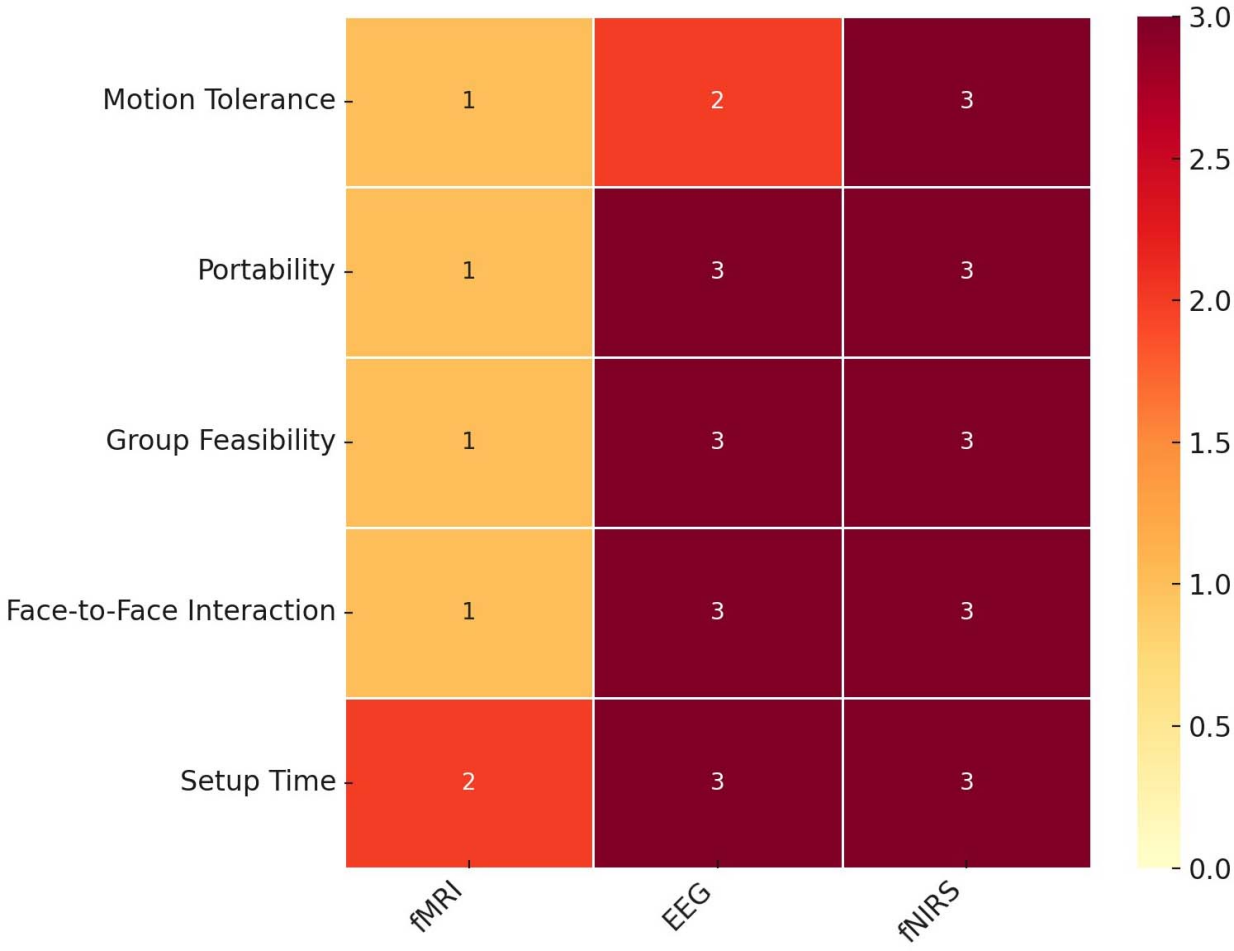
Suitability of neuroimaging technologies for hyperscanning studies of social interaction. A Heatmap comparing three neuroimaging technologies - functional Magnetic Resonance Imaging (fMRI), electroencephalography (EEG), and functional near-infrared spectroscopy (fNIRS) - across five metrics relevant to social interaction studies: motion tolerance, portability, group feasibility, face-to-face interaction capability, and setup time. Each metric is scored on a scale from 0 (least suitable) to 3 (most suitable). Darker shades indicate higher suitability within each metric. fMRI shows limitations in motion tolerance and portability, while EEG and fNIRS demonstrate higher suitability in enabling face-to-face interactions and group setups. Both EEG and fNIRS have faster setup times relative to fMRI.

Studies post-2002 continued to utilize dual fMRI scanners to conduct hyperscanning studies (Tomlin, 2006; Saito et al., 2010; Tomlin et al., 2013; Spiegelhalder et al., 2014; Koike et al., 2016, 2019). However, despite the outstanding spatial resolution offered by fMRI, its integration into hyperscanning did not gain widespread traction. This is potentially due to the inherent constraints of scanners, limiting participants’ ability to move and communicate. These factors not only limit the range of questions that can be explored but also cast doubt on the ecological validity of the obtained results (Burgess, 2015). Consequently, fNIRS and EEG emerged as powerful alternatives. The allure of these methods lies in their portability and flexibility, allowing participants to move freely and engage in settings that mimic real-world scenarios (Figure 1). Indeed, these methods have now been utilized to capture neural activity across various social activities such as musical performances and classroom interactions (Zamm et al., 2021; Dikker et al., 2017).

The adaptability of fNIRS and EEG becomes particularly significant when investigating the effects of environmental conditions on the neural dynamics of social interactions. These advanced technologies provide unprecedented opportunities to ask, and answer, questions that were previously inaccessible due to technological limitations. For the first time, researchers can bridge behavioral insights with neural mechanisms to explore how environmental factors, such as, sound, lighting, proximity, and virtual reality, influence IBC.

Although there is a growing recognition of the importance of environmental factors in shaping social behavior, the application of hyperscanning technologies to study environmental modulation of IBC remains in its infancy. This limited exploration offers fertile ground for establishing new paradigms that examine the intricate interplay between environmental factors and social dynamics.

Despite this potential, there is a distinct absence of reviews that comprehensively synthesize and critically evaluate the body of literature dedicated to this emerging trend. Addressing this gap forms the rationale for the present review, which aims to position the environmental modulation of IBC as a critical new area of inquiry. Far from being a mere summary, this synthesis establishes a foundation for a novel research domain that integrates behavioral and neural perspectives to explore the induction effects of environmental conditions on the social brain. In this context, environmental conditions refer to external factors present within experimental settings.

Specifically, this review (1) examines the diverse protocols employed, (2) investigates the range of environmental conditions explored, and (3) elucidates the modulation of IBC in response to environmental change. Ultimately, this effort seeks to identify both the opportunities and challenges that define the research landscape in this evolving field and inspire further research leveraging advanced technologies to address critical questions relating to the interplay between our environments and social interactions.

## 2 Methods

The review followed the Preferred Reporting Items for Systematic Reviews and Meta-Analyses (PRISMA) guidelines (Liberati et al., 2009). Studies investigating the impact of changes in the environment on IBC during social interaction were discovered through searches on PubMed and Scopus, using the search terms: (social decision making OR social learning OR social reward OR social feedback OR peer feedback OR social norm OR social interaction OR social relationship OR interpersonal interaction OR interpersonal relationship OR social influence OR social information OR social bonding OR cooperation OR competition OR conflict OR communication OR altruis* OR trust OR reciprocity OR reputation OR social approval OR social status OR social hierarchy OR social exclusion OR social acceptance OR social preference OR social conformity) AND (hyperscanning OR two-person neuroscience OR interbrain OR interbrain-coupling) AND (environment OR environmental factors OR environmental changes OR environmental modifications OR sound OR noise OR music OR volume OR odor OR odor OR scent OR smell OR fragrance OR perfume OR light OR lighting OR brightness OR surroundings OR temperature OR stress) AND NOT (brain-computer interface OR BCI). Terms were searched for in paper titles and abstracts only. Filters were then applied so only published, original, research articles, in the English language, from 2000 to February 2025, were considered. During the exploratory phase of the review, additional databases were also searched, including Web of Science, Embase, and PsycINFO. However, these yielded substantial overlap with Scopus and did not return any additional eligible studies. Therefore, Scopus and PubMed were retained as the primary sources due to their combined coverage of biomedical, psychological, and interdisciplinary research. Preprint servers (e.g., arXiv, bioRxiv) were not included, as the focus of this review was on peer-reviewed, full-text empirical articles.

The primary search resulted in a total of 106 documents. Twenty-five documents were excluded because they were review papers, meta-analyses, conference papers, book chapters, meeting abstracts, notes, surveys, non-English language publications, or lacked full-text access. In line with the PRISMA recommendation, the PICOS (Population, Intervention, Comparator, Outcomes, Study designs) eligibility criteria were defined (Table 1). We exclusively considered studies involving healthy, neurotypical human participants, spanning all age groups and genders. Our inclusion criteria mandated the use of hyperscanning techniques to observe interactions between two or more individuals while manipulating environmental factors such as lighting, noise, music, or other contextual elements. The required outcome measured was IBC and hence the defined comparator was how IBC varies during interactions under different environmental conditions.

**TABLE 1:** PICOS eligibility criteria.

Criteria	Requirements
Population	Healthy human participants interacting and scanned simultaneously
Intervention	Functional Near-Infrared Spectroscopy (fNIRS), Electroencephalography (EEG), Functional Magnetic Resonance Imaging (fMRI), Magnetoencephalography (MEG), or any combination of these techniques used to simultaneously scan two or more brains during a social interaction involving an environmental change.
Comparators	Environmental change compared with control or different levels of environmental change
Outcomes	Inter-brain dynamic analysis
Study Design	Standard cognitive neuroscience protocols

Studies investigating the impact of stress on IBC during social interaction were not included, as such studies only resulted in changing the psychological state of participants before they undertook a task, rather than changing the environment in which the social task was conducted.

Many studies have examined the relationship between music and IBC, as reviewed in Cheng et al. (2024). However, the majority of these studies were excluded because they did not meet our inclusion criteria. Specifically, we excluded studies where music functioned as the primary social task, for example, in musical collaborations such as singing/instrumental duets, quartets, drumming games, or music therapy. In these cases, music is not an environmental context modulating a distinct social interaction, but rather constitutes the social interaction itself, making it difficult to disentangle the effects of music from the social behavior being studied. In contrast, our review focuses on studies where music acts as an exogenous environmental factor that modulates an existing or ongoing social behavior (e.g., conversation, storytelling, joint attention). This conceptual distinction is central to our review’s scope: we aim to examine how external environmental inputs, such as auditory, visual, or spatial factors, influence the neural dynamics of social interaction, rather than how IBC arises from tasks inherently based on musical coordination.

The study by Chabin et al. (2022), examining how IBC between audience members is affected by (1) music and (2) interpersonal distance was included because, although there is no direct social interaction between audience members, they share a social experience by attending a concert together.

Finally, the studies by Li et al. (2021, 2022) investigating IBC while participants listened to a recorded story told by another individual were included because although there was no live interaction, the study still captured interaction dynamics between the storyteller and the listener through IBC measurements. This approach aligns with our inclusion criteria as it examines brain coupling during a social behavior (storytelling) in response to an environmental stimulus (background noise).

**FIGURE 2:**
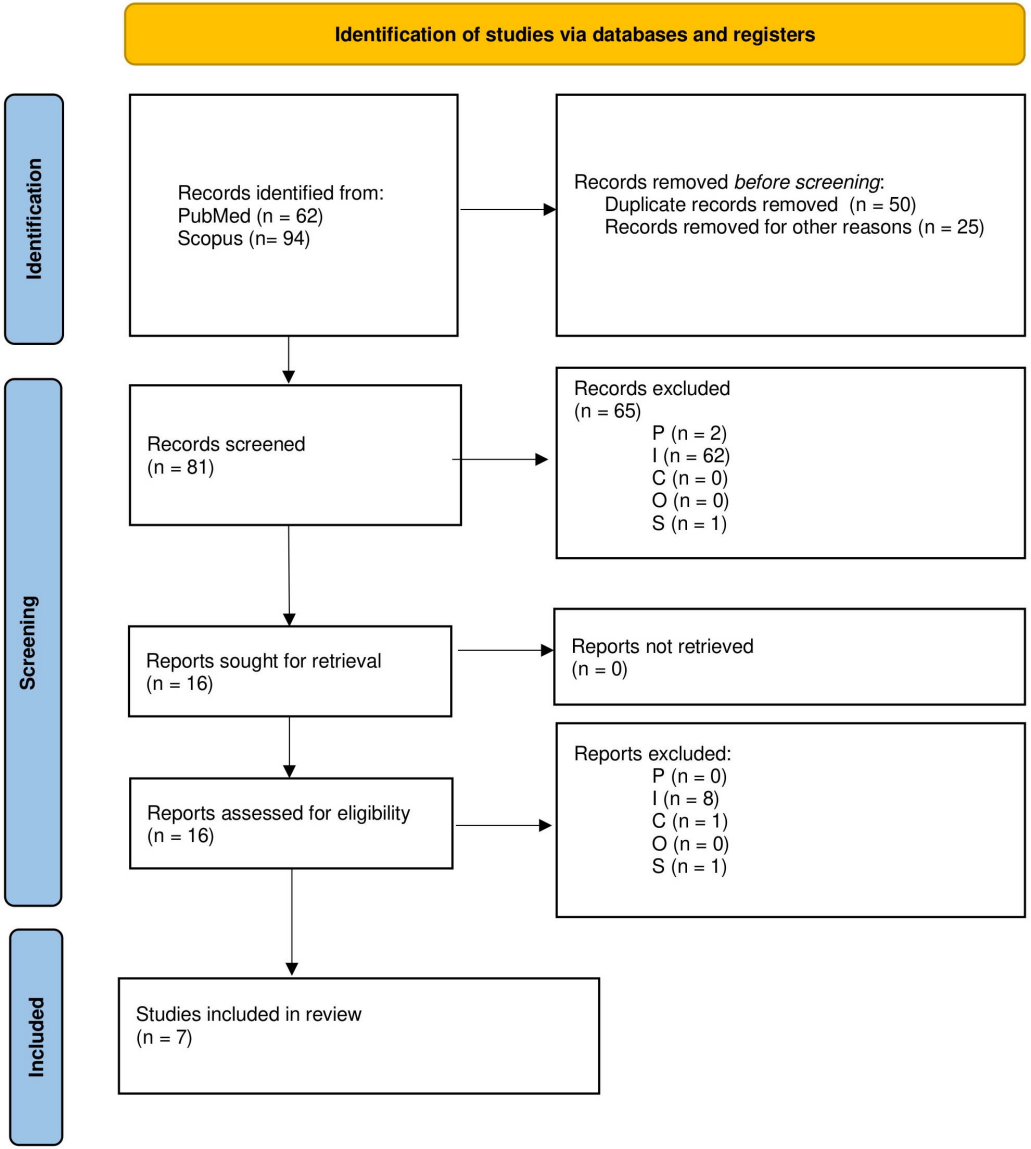
Preferred Reporting Items for Systematic Reviews and Meta-Analyses (PRISMA) flowchart depicting the selection process of studies included in the review.

The PICOS eligibility criteria from Table 1 were first applied to the title and abstracts of the remaining 81 papers, and a further 65 papers were removed (P: 2, I: 62, S:1). After full-text screening a further nine were removed (I:8, C:1). Many papers were removed because they did not meet the intervention criteria, i.e., they did not investigate or implement a change in an environmental condition. The PRISMA flowchart is shown in Figure 2. After screening, seven papers remained for analysis (Gumilar et al., 2021; Li et al., 2021; Chabin et al., 2022; Hu et al., 2022; Khalil et al., 2022; Li et al., 2023; Pan et al., 2023).

A formal risk of bias (RoB) tool was not used because none were found to be suitable for the types of studies included in this review. Existing tools assume features like randomization, blinding, structured interventions, or predefined clinical outcomes, none of which align with the structure of the IBC research presented. Instead, we developed a custom RoB assessment framework tailored to the context of the reviewed studies. Our tool evaluated seven domains tailored to hyperscanning research: (1) participant selection, (2) task design validity, (3) EEG/fNIRS measurement quality, (4) synchrony analysis and metrics, (5) stimulus/environmental control, (6) transparency of outcome reporting, and (7) interpretation bias. Each study was assessed independently across these domains and rated as low risk, some concerns, or high risk, with justifications provided. This approach ensured a structured, transparent, and context-appropriate evaluation of study quality.

## 3 Results

### 3.1 Population, study design and experimental paradigms/protocols

**FIGURE 3:**
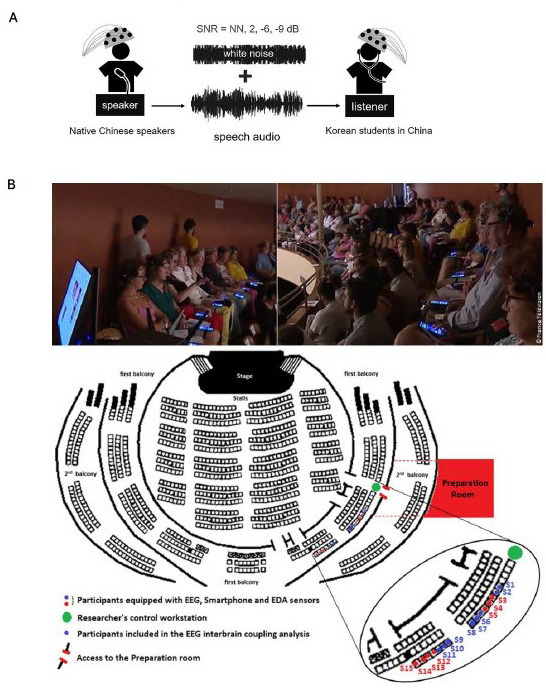
The experimental set-up of two studies reviewed. **(A)** Speaker participants recording storytelling audio while functional near-infrared spectroscopy (fNIRS) is recorded. Various levels of background noise were added to the audio, which was later played to listener participants while fNIRS was recorded. SNR, signal-to-noise ratio; NN, no noise (Reproduced with permission, Li et al., 2023). **(B)** Participants in the audience watching a concert while EEG is recorded (Reproduced with permission, Chabin et al., 2022).

The studies reviewed share similar participant demographics but employ distinct experimental paradigms to investigate interpersonal dynamics (Table 2; Figure 3). Coordination and imitation were explored through finger-tapping (Khalil et al., 2022; Hu et al., 2022) and finger-tracking exercises (Gumilar et al., 2021), while storytelling interactions examined native and non-native speech comprehension (Li et al., 2021, 2023). In the native context, both speaker and listener were Chinese natives, whereas in the non-native study, Chinese speakers were paired with Korean listeners proficient in Chinese. Chabin et al. (2022) simulated a RW concert experience, while Pan et al. (2023) engaged participants in collaborative design tasks. In contrast to prior studies that primarily focused on dyadic interactions, Chabin et al. (2022) included 15 participants, whereas Pan et al. (2023) formed groups of three but recorded neural activity from only two members in each group.

**TABLE 2:** A summary of the populations studied in the reviewed articles, along with an overview of the experimental procedures used.

Paper	Population (*n* = number of participants; sex; age range in years)	Experimental protocol			
		Social Task	Environmental change	Conditions	Study design
Gumilar et al. (2021)	Healthy adults (*n* = 24; 19 Male, 5 Female; ages not specified)	Finger-pointing exercise separated by a finger-tracking exercise	Virtual Reality (VR)	Real-world (RW): Face-to-face VR: Face-to-face VR: First person perspective	Pre-training - sat facing each other, extended one arm with the index finger pointing out, looked at each other’s fingertips (1 min/arm) Training - participants followed the fingertip of the designated leader, attempting to mimic the movements. Repeated for both arms, and the roles of leader and follower were switched (6 min) Post-training - repeated the pre-training finger pointing task (1 min/arm)
Li et al. (2021)	Healthy adults (*n* = 22; 11 Male, 11 Female; 18–25 years)	Story-telling	Background noise	Rest Audio 1 (no noise level) Audio 2 (signal to noise ratio = 2 dB) Audio 3 (signal to noise ratio = -6 dB) Audio 4 (signal to noise ratio = -9 dB)	32 trials (8 trials for each of the 4 noise levels) Rest (3 min) Listen (1.5 min) Clarity and Intelligibility rating and Comprehension test Break (20 s)
Chabin et al. (2022)	Healthy adults (*n* = 15; 3 Male, 12 Female; 18–78 years)	Participants watched six conductors in turn conducting a full symphonic orchestra and choir, performing extracts	Distance from each other and Music	Baseline (no music played by the orchestra) Music	Participants seated in the first balcony of a concert hall on a single row of seats. Participants watched six conductors in turn conducting a full symphonic orchestra and choir, performing extracts. Each performance lasted approximately 25 min, including repetitions and instructions to musicians.
Khalil et al. (2022)	Healthy adults and 1 child (*n* = 10; 3 Male, 7 Female; 8–50 years)	Anti-phase tapping game	Music	Pre tapping game Post tapping game	Rest (sitting in silence, not looking at each other) (2 min) Anti-phase tapping game (5–10 min) Rest (sitting in silence, not looking at each other) (2 min)
Hu et al. (2022)	Healthy adults (*n* = 40; 0 Male, 40 Female; 18–25 years)	Finger-tapping task	Musical meter	Musical Meter/received the auditory feedback of partner’s response No Meter/received the auditory feedback of partner’s response Musical Meter/received the auditory feedback of own response No Meter/received the auditory feedback of own response	4 Blocks (one of each condition), 30 s rest between blocks Rest (20 s) Instruction (3 s) Stimulus (Meter/No Meter) (12 s) Tapping (15 trials/block) (12 s)
	Healthy adults (*n* = 32; 0 Male, 32 Female; 18–28 years)			Strong Meter/Duple Frequency/received the auditory feedback of partner’s response Strong Meter/Triple Frequency/received the auditory feedback of partner’s response Weak Meter/Duple Frequency/received the auditory feedback of partner’s response Weak Meter/Triple Frequency/received the auditory feedback of partner’s response	4 Blocks (one of each condition), 30 s rest between blocks Rest (2 min) Instruction (3 s) Stimulus (Strong Meter/Weak Meter) (12 s) Tapping (8 trials/block) (12 s)
Li et al. (2023)	Healthy adults (*n* = 21; 9 Male, 12 Female; 18–25 years)	Story-telling	Background noise	Rest Audio 1 (no noise level) Audio 2 (signal to noise ratio = 2 dB) Audio 3 (signal to noise ratio = -6 dB) Audio 4 (signal to noise ratio = -9 dB)	32 trials (8 trials for each of the 4 noise levels) Rest (3 min) Listen (1.5 min) Clarity and Intelligibility rating and Comprehension test Break (20 s)
Pan et al. (2023)	Healthy adults (*n* = 30; 12 Male, 18 Female; mean 23.5 ± 1.2 years (range not specified)	Collaborative design tasks	Virtual Reality	RW Virtual Reality	Rest (1 min) Idea Generation Phase (2 × 5 min blocks) Rest (1 min) Idea Selection Phase (2 × 5 min blocks) Rest (1 min) Idea Deepening Phase (2 × 5 min blocks) Rest (1 min)

Study designs and environmental conditions varied (Table 2). Finger-tapping (Khalil et al., 2022; Hu et al., 2022) and storytelling studies (Li et al., 2021, 2023) used block designs with rest periods, testing variables such as music and background noise levels, respectively. In contrast, Gumilar et al. (2021), Chabin et al. (2022), and Pan et al. (2023) all used within-subject designs, with Gumilar et al. (2021) and Pan et al. (2023) comparing VR and real-life conditions, while Chabin et al. (2022) focused on the effects of interpersonal distances and music in a concert setting.

### 3.2 Data acquisition

Four studies reviewed employed fNIRS (Li et al., 2021; Hu et al., 2022; Li et al., 2023; Pan et al., 2023), while the other three utilized EEG (Chabin et al., 2022; Gumilar et al., 2021; Khalil et al., 2022; Table 3). All fNIRS studies utilized the optimal source-detector distance of 30 mm, allowing for the interrogation of the cortical areas of the brain, except for Pan et al. (2023) who did not specify the source-detector distance used.

**TABLE 3:** Summary of the imaging devices and data acquisition features.

Paper	Data acquisition							
	Imaging modality	Device	Company/ country	Wavelengths	Number of channels	Source-detector separation	Cortical brain region	Sampling frequency
Gumilar et al. (2021)	Electroencephalography (EEG)	g.GAMMAsys	g.tec medical engineering GmbH, Austria	N/A	16	N/A	Frontal, central, parietal, temporal and occipital lobes	512 Hz
Chabin et al. (2022)	EEG	Emotiv EPOC	Emotiv, USA	N/A	14	N/A	Frontal, central and parietal lobes	128 Hz
Li et al. (2021)	Functional Near-Infrared Spectroscopy (fNIRS)	NirScan Inc.	HuiChuang, Beijing	785, 808, and 850 nm	36	30 mm	Prefrontal, parietal, temporal and occipital cortex	12 Hz
Khalil et al. (2022)	EEG	Practical Mobile Dry EEG System	Cognionics	N/A	5	N/A	Frontal and occipital lobe and sensorimotor area	4,000 Hz
Hu et al. (2022)	fNIRS	ETG-7100 optical topography system	Hitachi Medical Corporation, Japan	780 nm and 830 nm	Exp 1: 22 Exp 2: 44	30 mm	Exp 1: Frontal cortex (including the motor and premotor areas) Exp 2: Frontal cortex (including the motor and premotor areas) and Prefrontal cortex	10 Hz
Li et al. (2023)	fNIRS	NirScan Inc.	HuiChuang, Beijing	Listeners: 740 and 850 nm Speakers: 785, 808, and 850 nm	36	30 mm	Prefrontal,parietal, temporal and occipital cortex	Listeners: 17 Hz Speakers: 12 Hz
Pan et al. (2023)	fNIRS	Nirsmart	Danyang Huichuang Medical Equipment Co. Ltd., China	Not specified	25	Not specified	Left Prefrontal Cortex (l-PFC), Left Temporoparietal Junction (l-TPJ), and Left Premotor Cortex (l–PMC)	Not specified

Most studies recorded brain activity simultaneously from all individuals within a dyad, or from all 15 participants during the concert scenario (Chabin et al., 2022; Gumilar et al., 2021; Hu et al., 2022; Khalil et al., 2022; Pan et al., 2023). However, two studies investigating the impact of background noise, took a different approach (Li et al., 2021, 2023). In these instances, ‘each dyad consisted of a speaker and a listener. The speaker verbally narrated a story, which was recorded, while their brain activity was monitored. These recordings were then manipulated, by adding various levels of white noise to create four signal-to-noise ratio (SNR) conditions. Subsequently, the recorded audios were played back to the listener while their brain activity was recorded.

The integration of additional physiological metrics was uncommon among the studies surveyed. Only one study reported taking measures such as heart rate and skin conductance, using the external device Shimmer^®^ Sensing GSR + (Chabin et al., 2022).

### 3.3 Optode placement and channel configuration

All investigations highlighted the frontal lobe’s role in decision-making, emotional regulation, motor planning, and social cognition, with most studies also focusing on the parietal lobe for spatial awareness and sensorimotor integration, and several examining the occipital and temporal lobes for visual processing, auditory processing, and language comprehension (Table 3).

Multichannel configurations play a crucial role in capturing the spatial distribution and dynamics of neural signals across different brain regions. Higher channel density provides finer spatial resolution but may also increase complexity and data processing requirements. All studies reviewed opted to use multichannel probe designs (Table 3). EEG studies used between 5 and 16 channels (Chabin et al., 2022; Gumilar et al., 2021; Khalil et al., 2022), while the fNIRS studies used between 22 and 36 channels (Li et al., 2021; Hu et al., 2022; Li et al., 2023; Pan et al., 2023). It is typical for EEG to employ fewer channels as the scalp’s conductive properties enable EEG signals to be detected by electrodes without requiring a high channel density. The EEG studies also used higher sampling frequencies (128–4,000 Hz) compared to the fNIRS studies (10–17 Hz). This is because EEG directly measures electrical activity in the brain, which fluctuates rapidly. On the other hand, fNIRS measures hemodynamic responses, which change more slowly, allowing for lower sampling frequencies.

Ensuring consistent positioning of optodes over targeted brain regions across subjects is essential to guarantee reliable and comparable data, as slight variations in placement can lead to differences in the brain regions being measured. To achieve this, all studies, except for one (Khalil et al., 2022), utilized the 10–20 system for electrode placement (Jasper, 1958; Okamoto and Dan, 2005). The 10–20 system maps out specific anatomical landmarks on the scalp based on the percentages of the distance between four key reference points: the nasion, inion, and the left and right preauricular points. In addition to employing the 10–20 reference system, 3 fNIRS studies specified further procedures to ensure accurate localization of cortical areas (Li et al., 2021; Hu et al., 2022; Li et al., 2023). One method involved the projection of topographic data onto a 3D reference frame based on skull landmarks, thereby allowing a probabilistic reference to cortical regions underlying each channel (Hu et al., 2022). Other studies employed virtual registration methods to establish correspondence between fNIRS channels and measurement points on the cerebral cortex (Li et al., 2021, 2023). By utilizing a 3D digitizer for precise channel positioning, researchers obtained potential coordinates for each channel. Subsequently, brain regions associated with the channels were determined using the Automated Anatomical Labeling (AAL) atlas (Tzourio-Mazoyer et al., 2002).

### 3.4 Pre-processing

In reviewing the preprocessing methods applied across the studies, several commonalities and distinctions emerged. While all studies prioritized the removal of motion artifacts to enhance the quality and reliability of the data, the specific techniques employed varied (Table 4).

**TABLE 4:** Summary of the steps adopted for the fNIRS and EEG data pre-processing.

Paper	Data pre-processing		
	Artifact correction	Filtering	Additional steps
Gumilar et al. (2021)	Independent component analysis (ICA)	0.5–60 Hz band-pass filter Notch filter	The worst two identified artifact components were rejected. The remaining components, which were considered clean, were used to reconstruct the Electroencephalography (EEG) signals
Li et al. (2021)	Targeted Principal Component Analysis (tPCA), hmrMotionArtifactByChannel Function, corrected by a cubic spline interpolation method	None specified	None specified
Chabin et al. (2022)	Principal Component Analysis (PCA) for regression of ocular artifacts Visual inspection of data cut into 1s periods	1–30 Hz low- and high-pass Butterworth filter 50 Hz notch filter	After regression of ocular artifacts, data were resynchronized and down-sampled to 125 Hz based on timestamps.
Khalil et al. (2022)	Overlapping sections of EEG data free from eyeblink and EMG (i.e., clean in both participants at the same time in the recording) were identified	0.5 to 60 Hz Finite Impulse Response (FIR) band-pass filter	Temporal alignment of the two EEG files from a participant dyad using recorded triggers
Hu et al. (2022)	The correlation-based signal improvement method	None specified	None specified
Li et al. (2023)	tPCA, hmrMotionArtifactByChannel Function, corrected by a cubic spline interpolation method	None specified	Data of the listeners were down-sampled to 12 Hz to match the sampling rate of the speakers
Pan et al. (2023)	None specified	0.01–0.2 Hz Butterworth filter	None specified

Gumilar et al. (2021) employed independent component analysis (ICA) to address multiple artifact types simultaneously, an approach that performs particularly well in the non-Gaussian signal environments typical of EEG data. However, ICA is computationally intensive and generally more effective with larger datasets, so the relatively modest dataset size in this study (16 channels) may have limited its effectiveness. In contrast, Chabin et al. (2022) applied the more computationally efficient principal component analysis (PCA), which removes components explaining the most variance, usually artifacts. PCA’s faster processing makes it better suited to smaller datasets, such as the 14 channels used, though it is more prone to overcorrection and struggles to differentiate between artifacts and meaningful neural signals as effectively as ICA.

Building on the advantages of PCA, targeted principal component analysis (tPCA) was applied by Li et al. (2021, 2023) alongside cubic spline interpolation. This adaptation of PCA is designed to more precisely isolate and remove motion artifacts by focusing on predefined artifact periods, reducing the risk of overcorrection seen in traditional PCA. Given both studies’ relatively high number of channels (36), tPCA was particularly well-suited, allowing for effective artifact reduction without excessive data loss. However, like PCA, tPCA relies on the accurate identification of artifact epochs, which can introduce bias if artifacts are misclassified. Additionally, tPCA can be time-consuming due to the manual selection of parameters.

In contrast, Hu et al. (2022) utilized a correlation-based signal improvement method, which targets the preservation of hemoglobin signal changes by addressing motion-induced correlations between HbO2 and Hb. While this method is effective for neurotypical populations, it is less suited to populations with atypical brain physiology, as it assumes a consistent negative correlation between HbO2 and Hb, limiting its generalizability to more diverse populations (Cui et al., 2010; Obrig and Steinbrink, 2011). Given Hu et al.’s use of more than 20 channels, this method allowed for a robust analysis of hemoglobin signal changes across a larger dataset.

Manual artifact removal, as used by Khalil et al. (2022), allows for human oversight in distinguishing artifacts from neural signals. This method is particularly useful when artifacts are difficult to identify through automated processes. However, manual selection is subjective, time-consuming, and difficult to replicate consistently across datasets and researchers. Given the small scale of this study (only five channels) manual selection was a more appropriate choice, as using ICA or PCA would likely have been ineffective with such limited data.

Filtering was another key preprocessing step employed by four studies (Pan et al., 2023; Chabin et al., 2022; Gumilar et al., 2021; Khalil et al., 2022). In fNIRS, filters are essential for removing physiological artifacts, such as cardiac pulsations and respiratory signals, without altering the functional hemodynamic response. Similarly, in EEG, filters help eliminate low-frequency drift, muscle artifacts, and electrical noise to ensure cleaner neural signals. The choice of filters varied among the studies, depending on the specific noise characteristics and research focus. A detailed review of the advantages and limitations of various filtering techniques is available in Dans et al. (2021).

Both Chabin et al. (2022) and Khalil et al. (2022) also employed data alignment procedures to ensure temporal synchronization across participants. This step is crucial for group-level analyses, particularly in complex neuroimaging studies where even minor misalignments can distort time-sensitive neural response interpretations.

### 3.5 Analysis

All reviewed fNIRS studies employed Wavelet Transform Coherence (WTC) analysis to measure IBC (Li et al., 2021; Hu et al., 2022; Li et al., 2023; Pan et al., 2023) (Table 5). WTC decomposes time-varying signals into different frequency bands, calculating coherence between the corresponding frequency components of signals from different brains. This method provides an estimate of the degree of synchrony across participants’ brain signals under various experimental conditions. However, while WTC is widely used in fNIRS research (Hakim et al., 2023), it poses certain challenges. One of the main limitations is the ambiguity around the selection of frequency bands in fNIRS, as the physiological meaning of different frequency components is less clear compared to more direct neural measures like EEG. fNIRS signals often include a combination of physiological processes unrelated to cognitive activity, making it difficult to directly associate frequency bands with brain function. Current methods for selecting frequency bands, whether through visual inspection, task-related associations, or significance testing, are often lacking in mechanistic justification. The studies could have strengthened their analyses by pre-registering frequency band selections or providing a stronger theoretical basis for their chosen bands.

**TABLE 5:** Overview of the analysis of fNIRS and EEG data.

	Data analysis				
Paper	Method to measure brain activation and IBC	Signal	Frequency of interest	Software/package	Statistical tests
Gumilar et al. (2021)	Electroencephalography (EEG) Source Localization	Current density in voxels	Delta 0.5–3.5 Hz Theta 4–7.5 Hz Alpha 8–11.5 Hz Beta 12–29.5 Hz Gamma 30–60 Hz	Statistical non-parametric mapping, LORETA-key software	None specified
	PLV	None specified	None specified	EEGLAB, MATLAB	Chi-squared analysis
Li et al. (2021)	WTC	HbO and HbR	0.01–0.7 Hz	Wavelet transform coherence (WTC), MATLAB	Repeated measures One-way Analysis of Variance (ANOVA) with a non-parametric cluster-based permutation method
Chabin et al. (2022)	Total Interdependence (TI) and theta coherence (ThetaCo)	None specified	TI 1–20 Hz ThetaCo 4–8 Hz	None specified	Wilcoxon signed-rank tests and Friedman tests for comparing TI and ThetaCo indices across different conditions *Post hoc* pairwise Durbin–Conover tests were used for specific comparisons following ANOVA Spearman’s correlations were performed to explore relationships between participant positions, EEG indices, subjective emotional reports, and individual trait measures
Khalil et al. (2022)	Wavelet analysis and cross-correlation	None specified	Delta 0.5–3 Hz Theta 4–8 Hz	None specified	Linear mixed effects model Paired sample *t*-tests
Hu et al. (2022)	Wavelet transform coherence (WTC), Cross-correlation	Oxygenated hemoglobin (HbO) and deoxygenated hemoglobin (HbR)	0.015–0.1 Hz	WTC, HERMES, MATLAB	A cluster-based permutation test
	WTC	HbO and HbR	0.015–0.1 Hz	WTC, HERMES, MATLAB	A cluster-based permutation test with Linear mixed-effects model
Li et al. (2023)	WTC	HbO and HbR	0.01–0.032 Hz	WTC, MATLAB	Repeated measures One-way Analysis of Variance (ANOVA) with false discovery rate (FDR) correction
Pan et al. (2023)	WTC	HbO and HbR	0.047641–0.050474 Hz	WTC, MATLAB	Permutation test using pseudo-random pairing Paired sample *t*-tests ANOVA Pearson’s correlation

In contrast, two EEG studies employed phase-based methods to measure IBC, with each method tailored to the unique demands of the experimental task. Khalil et al. (2022) used Phase Correlation, a technique measuring the alignment between the phases of EEG signals in near-real time. This method was particularly suited for the fast-paced, dynamic nature of their anti-phase tapping game. Phase Correlation’s sensitivity to these moment-to-moment shifts made it the ideal choice for capturing immediate neural alignment, although it is less capable of measuring stable, sustained synchrony over longer periods, which might be important for post-task analyses. On the other hand, Gumilar et al. (2021) employed Phase Locking Value (PLV), which is better suited for measuring the consistency of phase alignment over multiple trials or longer periods. Their finger-pointing and tracking exercise involved repetitive, predictable actions, and PLV’s focus on long-term phase stability allowed the researchers to capture how consistently participants’ brain waves remained in sync throughout the task. While PLV is ideal for structured, repetitive interactions, it is less sensitive to transient changes in synchrony, which could be important in more dynamic or spontaneous tasks.

Chabin et al. (2022) took a different approach by using Total Interdependence (TI) and Theta Coherence (ThetaCo) to analyze IBC during a live concert setting. TI was computed to assess overall cerebral coupling between participants, accounting for feedback beyond concurrently recorded data points, which provided a more comprehensive measure of synchrony compared to traditional coherence. However, TI’s assumption of Gaussian stationarity may be problematic in dynamic settings like a concert, where brain signals are likely non-stationary. Additionally, the study used ThetaCo to focus specifically on inter-brain coherence in the theta frequency band (4–8 Hz), which is often associated with emotional processing. This allowed the researchers to capture neural synchrony related to shared emotional experiences, such as moments of high pleasure during the concert.

In addition to IBC, several studies also conducted single brain analyses to explore intra-brain activations. Li et al. (2021, 2023) analyzed the activity patterns within individual brains to understand how different regions interacted during task and rest periods, using statistical techniques like ANOVA, cluster-based permutation tests, and Pearson’s correlation. Gumilar et al. (2021) extended their analysis by incorporating EEG source localization, which mapped neural activity onto specific brain regions, providing spatial insight into the brain areas involved in IBC.

### 3.6 IBC, behavior and the environment

**FIGURE 4:**
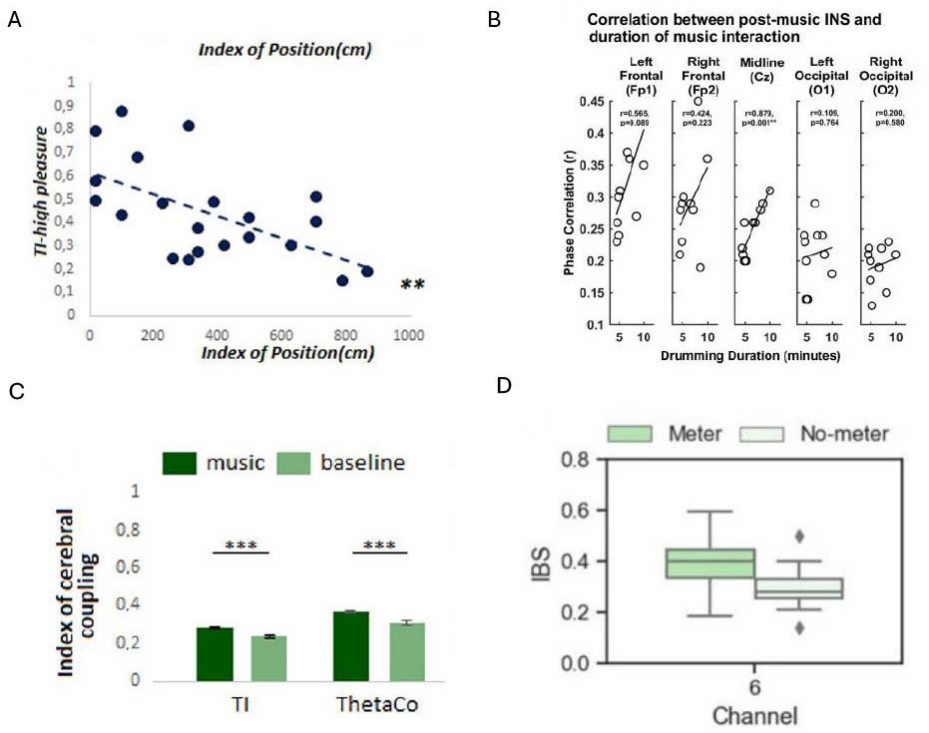
Examples of results displaying the impact of environmental factors on inter-brain coupling (IBC). **(A)** Pearson correlation showing the relationship between participants’ relative positions (distance between participants) and Theta inter-brain coupling (TI) indices during instances of reported high emotional synchrony (**P < 0.01) (Reproduced with permission, Chabin et al., 2022). **(B)** Correlations between the duration of musical interaction and delta band IBC in the POSTcondition. INS, interpersonal neural synchrony (Reproduced from Khalil et al., 2022, licensed under CC BY 4.0). **(C)** Comparison of TI and ThetaCo indices during music listening versus baseline conditions (***P < 0.0001) (Reproduced with permission, Chabin et al., 2022). **(D)** Illustration of increased IBC following exposure to a musical meter (left) versus no meter (right). IBS, inter-brain synchrony (Reproduced from Hu et al., 2022, licensed under CC BY 4.0).

**FIGURE 5:**
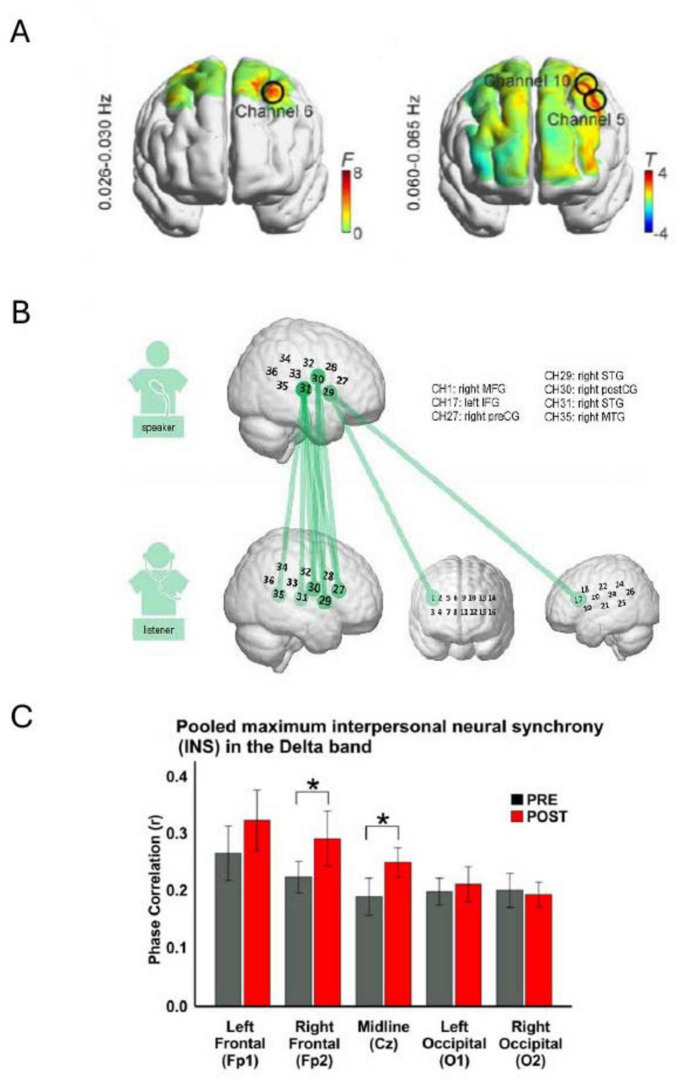
Illustrative results showing brain regions exhibiting inter-brain coupling (IBC). **(A)** Heat maps display IBC findings from two experiments: the left panel examines the contrast between musical meter and no-meter conditions, while the right panel compares strong versus weak meter conditions. Notably, higher IBC was detected at channels 5, 6, and 10 (Reproduced from Hu et al., 2022, licensed under CC BY 4.0). **(B)** Repeated measures ANOVA reveals speaker-listener neural coupling, where colored lines indicate channel combinations between the speaker and listener that show significant differences across the five conditions (*p* < 0.05, FDR corrected) (Reproduced with permission, Li et al., 2023). **(C)** Bar charts depict the average phase correlation in the PRE (gray) and POST (red) conditions across five electrode sites, with asterisks indicating statistically significant differences (*p < 0.05) (Reproduced from Khalil et al., 2022, licensed under CC BY 4.0).

IBC, as observed across studies, appears to be influenced by changes in the environment such as exposure to music, variations in interpersonal distance, VR simulations, and background noise levels (Figure 4). Moreover, implicated brain regions associated with IBC span various cortical areas (Figure 5; Table 6).

**TABLE 6:** Overview of the effect of each environmental factor on IBC and behavior/emotion, and the brain regions implicated.

Paper	Social task	Environmental change	Brain regions effected	Effect on behavior/emotion	Effect on Inter-brain coupling (IBC)	Behavior–brain correlation
Gumilar et al. (2021)	Finger-pointing exercise and finger-tracking exercise	Virtual reality (VR)	Dorsolateral Prefrontal Cortex (DLPFC), Middle Temporal Gyrus (MTG), Premotor Cortex (PMC), Somatosensory Association Cortex (SAC), Inferior Frontal Gyrus (IFG)	None specified	IBC increased in the real-world (RW) in theta and beta bands. In VR, IBC increased post-training in alpha and beta bands, with additional delta activity in the first-person perspective condition. Regions of interest (ROIs) included DLPFC, IFG, MTG, PMC, and SAC. Only ∼30% of ROIs in VR showed significant effects	None specified
Li et al. (2021)	Storytelling	Background noise	Angular Gyrus (AG), Left Superior Frontal Gyrus (l–SFG), Left Middle Frontal Gyrus (l–MFG), Left Supramarginal Gyrus (l–SMG), l–IFG, *r*-MTG	Comprehension, clarity, and intelligibility scores decreased significantly with increasing noise	l-IFG, SFG, MFG and SMG show declining IBC under noise. R-MTG and AG show stable IBC under noise	Stronger IBC in l- IFG predicts better performance under challenging (noisy) conditions
Chabin et al. (2022)	Watching a concert	Interpersonal Distance	Frontal, temporoparietal, and central regions (exact anatomical labels not reported)	Increased emotional sharing with music and closer distances	IBC increased with shorter distances during shared high pleasure	IBC increased significantly during high-emotion segments of the music compared to low-emotion segments
		Music			IBC increased in the presence of music	
Khalil et al. (2022)	An anti-phase tapping game	Music	Primary Motor Cortex/Primary Somatosensory Cortex (M1/S1), Right Prefrontal Cortex (*r*-PFC)	None specified	Delta-band IBC significantly increased POST interaction. No change in theta-band IBC	Longer shared musical engagement led to stronger IBC after the interaction
Hu et al. (2022)	Finger-tapping task	Musical meter	Left Middle Frontal Cortex (l–MFC)	Coordination increased in the presence of musical meter	IBC increased in the presence of musical meter	Higher IBC correlated with greater coordination accuracy between participants
Li et al. (2023)	Storytelling	Background noise	Right Precentral Gyrus (*r*-preCG), Right Superior Temporal Gyrus (r-STG), Right Postcentral Gyrus (r-postCG), r-MTG	Comprehension, clarity, and intelligibility scores decreased significantly with increasing noise	*r*-STG, postCG and MTG show greater IBC at higher noise (–9 dB), but not monotonic across all noise levels	Higher IBC in *r*-STG, r-postCG, and *r*-MTG correlated with better speech comprehension in noisy conditions
Pan et al. (2023)	Collaborative design tasks	VR	S1, l–PFC, STG, SMG, PMC/Supplementary Motor Area (SMA), Left Temporoparietal Junction (l–TPJ)	VR increased idea fluency, originality, and personal satisfaction, but sometimes at the cost of idea quality, especially in the early stages of collaboration	In VR, IBC was increased in the l–PFC and l–TPJ during idea selection, and in the S1 during idea deepening. In the RW, IBC was stronger in the PMC/SMA during idea generation, and increased in the l-PFC, S1, and STG during idea selection and deepening	Idea Quality: RW - Positively correlated with IBC in PMC/SMA and S1, VR - Negatively correlated with IBC in l-TPJ Originality: RW - positively correlated with IBC in l-TPJ and PMC, VR - positively correlated with IBC in l-PFC Fluency: RW - negatively correlated with IBC in l-TPJ

Gumilar et al. (2021) observed that while IBC can be achieved in VR environments, the degree of synchrony may differ compared to traditional face-to-face interactions, suggesting that visual perspective plays a crucial role in modulating neural coherence. In the real world (RW) scenario, IBC significantly increased from pre-to post-training in theta and beta bands. Notably, significant synchrony occurred in cortical regions such as the dorsolateral prefrontal cortex (DLPFC), the primary motor cortex (PMC), the middle temporal gyrus (MTG), the inferior frontal gyrus (IFG), and the somatosensory association cortex (SAC). Contrastingly, in VR, IBC significantly increased post-training in the alpha and beta bands, with additional delta-band activity in the first-person perspective condition. However, these effects were observed in only about 30% of regions of interest (ROIs). The results suggest that while there may be differences in the magnitude or patterns of synchrony, the fundamental mechanism of neural coupling remains intact across both virtual and real-life contexts.

Pan et al. (2023) extended these findings by investigating the effects of VR on IBC during collaborative design tasks. In the idea generation phase, participants in the RW setting demonstrated higher levels of IBC in areas such as the primary motor cortex (PMC) and supplementary motor area (SMA) compared to those in VR, likely due to the RW’s more immersive and tactile nature, better supporting motor and sensory integration. However, during the idea selection phase, VR participants exhibited greater IBC in the prefrontal cortex (PFC) and supramarginal gyrus (SMG). Increased activation in the PFC suggests the virtual environment may enhance cognitive synchronization and facilitate more efficient communication and decision-making. Additionally, VR fostered higher IBC in the left temporoparietal junction (l-TPJ), which is associated with creativity and idea evaluation, indicating that VR may support creative thinking processes. During the idea deepening phase, the VR environment led to greater IBC in the primary somatosensory cortex (S1), possibly due to the larger virtual space and enhanced tools that provided participants with a more stimulating environment for perception and engagement. On the other hand, the RW group displayed higher IBC in the superior temporal gyrus (STG), which plays a key role in social cognition and facial recognition, suggesting that RW interactions better facilitate implicit communication.

The results of the Chabin et al. (2022) study revealed a negative correlation between the proximity of participants and cerebral coherence indices, particularly evident when participants simultaneously reported high pleasure emotions when listening to music. This suggests that closer physical proximity fosters greater IBC implying a form of interindividual emotional influence. Interestingly, the study found no significant relationship between physiological coupling and proximity, suggesting distinct mechanisms underlying cerebral and physiological coherence. The findings also suggest that emotional resonance mechanisms may underlie the observed cerebral coupling, wherein shared emotional experiences lead to mutual oscillatory influences between individuals.

Khalil et al. (2022) investigated the effect of musical interaction on IBC and performance. They found a significant increase in delta band IBC after a musical interaction, particularly at electrodes over the central and right frontal brain areas. Notably, no significant theta-band IBC changes were reported. This suggests that musical interaction can influence brain dynamics at the interindividual level. Moreover, the duration of musical interaction was significantly correlated with delta-mediated IBC in the post-interaction condition, indicating a positive relationship between the duration of musical interaction and the degree of IBC during the interaction. In agreement with this, Hu et al. (2022) results suggest that exposure to a musical meter enhances IBC. IBC in the left middle frontal cortex (MFC), was significantly greater in the meter condition than in the no-meter condition. Moreover, IBC in the left MFC was higher in the presence of strong meters relative to weak meters. These results were further supported in the concert setting, as IBC was higher when people were listening to music compared with the baseline period (Chabin et al., 2022).

Li et al. (2021, 2023) found that background noise modulates IBC in distinct ways across cortical regions. In Li et al. (2021), IBC in left-lateralized language areas, specifically the IFG, Superior Frontal Gyrus (SFG), Middle Frontal Gyrus (MFG) and SMG, decreased as noise increased, whereas right MTG and AG showed stable IBC across noise levels. In contrast, Li et al. (2023) reported increased IBC in right-lateralized auditory-motor regions (*r*-STG, *r*-Postcentral Gyrus (postCG), and *r*-MTG) under the highest noise condition (−9 dB), though this increase was not monotonic across all noise levels. These findings suggest that while background noise can suppress coupling in traditional language areas, it may enhance compensatory coupling in auditory-motor networks under challenging listening conditions.

A noteworthy observation is the heterogeneous approaches adopted by researchers to present their findings on how environmental factors impact IBC and the associated brain regions (Figures 4, 5). This diversity in data visualization methods, ranging from bar graphs and scatter plots to box plots and detailed brain heat maps, reflects not only the varied nature of the investigations but also the challenges inherent in conveying this complex data. Such variability may underscore the necessity for standardized reporting formats in future research to facilitate comparison, synthesis, and meta-analytical assessments of findings across studies. Additionally, among the studies reviewed, only Pan et al. (2023) systematically reported effect sizes (Cohen’s d) across both behavioral and neural metrics, with medium to large effects (Cohen’s d = 0.55–1.63). The remaining studies generally reported statistical significance (*p*-values) but did not include standardized effect sizes, further limiting cross-study comparisons of magnitude.

Behavior and emotions also exhibited sensitivity to environmental factors such as music, distance, background noise and whether the environment was real or virtual. Closer physical proximity during a concert setting fostered greater emotional sharing among participants, suggesting a link between spatial distance and emotional resonance (Chabin et al., 2022). This was accompanied by increased IBC during high-emotion musical segments, indicating neural synchrony may underpin shared affective experiences. Moreover, the presence of a musical meter improved coordination during the finger-tapping task (Hu et al., 2022). Specifically, the mean interpersonal time lag was shorter in the meter condition. This behavioral improvement was positively correlated with higher IBC, suggesting that neural synchrony facilitates precise joint action. Conversely, Li et al. (2021, 2023) highlighted the detrimental impact of background noise on speech comprehension performance, with higher SNR levels resulting in lower comprehension performance. Interestingly, correlation analyses between speech comprehension performance and neural coupling suggested distinct behavioral relevance patterns for left and right-lateralized clusters (Li et al., 2021). In Li et al. (2021), stronger IBC in the l–IFG predicted better performance under high-noise conditions, while couplings in the right MTG and AG were more predictive of performance at mild noise levels. Similarly, Li et al. (2023) found higher IBC in the *r*-STG, *r*-postCG, and *r*-MTG was associated with better comprehension in noisy settings, highlighting compensatory auditory-motor coupling under degraded input. Finally, collaborative design behavior in VR and RW environments exhibited both similarities and differences (Pan et al., 2023). Although the overall quality of ideas generated by participants in VR and reality did not differ greatly, distinct patterns emerged at different stages of the design process. In the ideation phase, participants in VR tended to produce ideas of lower quality compared to those working in reality. However, they experienced a higher sense of fluency and subjective satisfaction. Additionally, during the elaboration phase, participants in VR expressed higher levels of satisfaction with their individual and team efforts compared to their RW counterparts. Interviews with participants suggested that VR tools fostered a more productive brainstorming process by generating more ideas than expected, despite some concerns about their quality. Notably, during the idea selection phase, participants in VR demonstrated greater originality, potentially due to the ease of communication and collaboration in a virtual setting. This was supported by positive correlations between originality and IBC in the l–PFC for the VR group, and in the l–TPJ and PMC for the RW group. Additionally, fluency in the RW group was negatively correlated with IBC in the l-TPJ, suggesting differing cognitive dynamics across settings.

### 3.7 Risk of bias assessment

A summary of the RoB assessment is presented in Table 7. Overall, all included studies were judged to have some concerns regarding RoB, primarily due to limited demographic diversity (e.g., college student samples), small sample sizes, and a lack of analytic techniques to disentangle true interpersonal neural coupling from common input or task-evoked synchrony. This is discussed further in sections 4.2.2 and 4.2.3.

**TABLE 7:** Risk of bias assessment of studies reviewed.

Paper	Participant selection	Task design validity	Measurement Quality (EEG/fNIRS)	Synchrony analysis and metrics	Context/Stimulus control	Outcome reporting	Interpretation bias	Overall risk
Gumilar et al. (2021)	Some Concerns: Participants were healthy adults, but limited demographic details and potential selection bias from university recruitment.	Low Risk: Participants completed comparable tasks in both VR and real world (RW) conditions, using a within-subjects design. The structure allowed for direct environment comparison.	Some Concerns: EEG with 16 channels. Used ICA for artifact removal, which is powerful but may be less effective with small EEG datasets. 10–20 placement was followed.	Some Concerns: Used Phase Locking Value (PLV), appropriate for measuring long-term synchrony. However, no controls for common input effects (e.g., no residual signals or shuffled-pair comparisons), limiting inference about true inter-brain coupling.	Low Risk: Both VR and RW tasks were carefully matched. Visual perspectives controlled (first-person vs. third-person), and training phases were standardized.	Low Risk: Behavioral and EEG outcomes were clearly reported. Regions of interest and synchrony patterns described with spatial detail.	Some Concerns: Interpretation of synchrony as social lacks critical control analyses (e.g., shuffled pairs, residual signal methods), increasing the likelihood that observed IBC reflects parallel processing rather than genuine interpersonal coupling.	Some Concerns
Li et al. (2021)	Some Concerns: Participants were right-handed, native Chinese speakers/listeners with normal hearing; inclusion criteria clearly reported. All participants were college students, limiting generalizability.	Some Concerns: Well-controlled listening task with real speech stimuli at varied noise levels. Participants gave ratings and completed comprehension questions after each narrative. Speaker and listener brain activity were recorded at different times. While this reduces mutual interaction confounds, it also limits real-time interpersonal synchrony assessment.	Low Risk: fNIRS with appropriate optode placement, source-detector distance (30 mm), and high-density (36-channel) configuration. Preprocessing included tPCA and spline interpolation.	Some Concerns: Used Wavelet Transform Coherence (WTC), suitable for fNIRS. However, frequency band choices lacked pre-registration or strong theoretical justification. No common input or task confound, as only listener’s were exposed to background noise, and speaker/listener roles were different.	Low Risk: Experimental manipulation (noise levels) was precise. Speaker-listener asymmetry (speaker recorded under clean audio, listener exposed to noise) avoids full shared-input confounds.	Low Risk: Clear reporting of comprehension outcomes and neural coupling results across noise levels. Analyses were transparent and well-aligned with stated aims.	Some Concerns: Interpretation of synchrony as social lacks critical control analyses (e.g., shuffled pairs, residual signal methods), increasing the likelihood that observed IBC reflects parallel processing rather than genuine interpersonal coupling.	Some Concerns
Chabin et al. (2022)	Some Concerns: Participants were self-selected from concertgoers who responded to an email invitation. Although inclusion criteria were clearly defined (e.g., right-handed, healthy adults, ticket holders, the final sample (*n* = 15) was skewed (mostly female, older adults, mix of musicians and non-musicians).	Low Risk: Emotional and neural synchrony was measured during a real event with live music. Naturalistic concert setting offers strong ecological validity.	Low Risk: EEG with 14 channels; preprocessing applied using PCA for motion artifact reduction. High sampling rate and synchronized recordings. Additional physiological data (EDA, heart rate) adds robustness.	Some Concerns: Used TI and Theta Coherence, suitable for emotional and neural synchrony. However, no controls for common input effects (e.g., no residual signals or shuffled-pair comparisons), limiting inference about true inter-brain coupling.	Low Risk: The stimulus (live orchestral music) was uniform for all participants in a specific session. Physical distance between participants was measured and incorporated into the analysis.	Some Concerns:Only a subset (15 of 37) participants were included in final analysis. Selection rationale was not fully explained.	Some Concerns: Interpretation of synchrony as social lacks critical control analyses (e.g., shuffled pairs, residual signal methods), increasing the likelihood that observed IBC reflects parallel processing rather than genuine interpersonal coupling.	Some Concerns
Khalil et al. (2022)	Some Concerns: Small sample of 10 participants recruited from a university audiology department. Mostly adults but included one child; no justification for age inclusion. Familiarity and prior knowledge of study aims varied; no matching for musical background.	Low Risk: Well-described, valid musical interaction (anti-phase tapping game). Pre/post design allowed for assessing change.	Some Concerns: Used only 5 EEG channels. Manual artifact rejection was appropriate for low channel count but subjective and non-replicable. Limited spatial resolution.	Some Concerns: Phase correlation used appropriately for dynamic motor task. Controlled for common task/input by measuring brain activity only before and after the tapping task, allowing them to attribute post-task IBC changes to the interaction.	Low Risk: Musical and non-musical blocks were clearly separated. Stimulus presentation (guide tones) was randomized	Low Risk: Reported both behavioral and EEG outcomes, including delta-band IBC increases and correlations with tapping duration.	Some Concerns: Measuring brain activity only before and after the tapping task, allowing them to attribute post-task IBC changes to the interaction itself, rather than to concurrent movement/motor synchrony.	Some Concerns
Hu et al. (2022)	Some Concerns: Female college students only, limiting generalizability. Gender selection was justified based on prior IBC literature. Participants were right-handed, unfamiliar with each other, and had minimal musical training.	Low Risk: Well-structured coordination and independence tapping tasks. Within-subject control for meter/no-meter stimuli. Block design with counterbalancing and rest periods.	Low Risk: Used fNIRS with appropriate source-detector distance and optode placement. 22 channels. Motion artifact correction via correlation-based method	Some Concerns: Used WTC, appropriate for fNIRS, but frequency band selection lacked mechanistic justification. However, no controls for common input effects (e.g., no residual signals or shuffled-pair comparisons), limiting inference about true inter-brain coupling.	Low Risk: Carefully designed auditory stimuli and real-time feedback. Participants seated without verbal/movement communication.	Low Risk: Reported IBC differences by condition and linked them with behavioral performance (tapping lag). Provided relevant statistical analyses.	Some Concerns: Interpretation of synchrony as social lacks critical control analyses (e.g., shuffled pairs, residual signal methods), increasing the likelihood that observed IBC reflects parallel processing rather than genuine interpersonal coupling.	Some Concerns
Li et al. (2023)	Some Concerns: Sample consisted of 15 right-handed, Korean female college students with fluent Chinese. Demographic homogeneity and cross-cultural background may limit generalizability. Sample size guided by prior studies.	Some Concerns:Well-controlled listening task with real speech stimuli at varied noise levels. Participants gave ratings and completed comprehension questions after each narrative. Speaker and listener brain activity were recorded at different times. While this reduces mutual interaction confounds, it also limits real-time interpersonal synchrony assessment.	Low Risk: Used 36-channel high-density fNIRS setup on both speaker and listener sides. Preprocessing applied (tPCA and spline), and spatial registration conducted using MNI projections.	Some Concerns: Used WTC, focusing on a pre-selected low-frequency band (0.01–0.032 Hz). Band was based on previous study. No common input or task confound, as only listener’s were exposed to background noise, and speaker/listener roles were different.	Low Risk: Narratives and noise levels were pre-recorded, standardized, and randomized. Resting-state data were collected. Participants were aware that the audios were pre-recorded.	Low Risk: Comprehension scores, intelligibility ratings, and WTC-based coupling metrics were clearly reported and statistically analyzed.	Some Concerns: Interpretation of synchrony as social lacks critical control analyses (e.g., shuffled pairs, residual signal methods), increasing the likelihood that observed IBC reflects parallel processing rather than genuine interpersonal coupling.	Some Concerns
Pan et al. (2023)	Some Concerns: Gender was matched and sample size was reasonable, but only 2 out of 3 group members were recorded with no justification for selection. All participants were college students, limiting generalizability.	Low Risk: Collaborative design tasks were clearly structured with distinct phases (ideation, selection, deepening). RW and VR conditions were well-matched.	Some Concerns: fNIRS used with high-density probe setup (22–36 channels), but source-detector distances were not reported.	Used Wavelet Transform Coherence (WTC), appropriate for fNIRS. However, no controls for common input effects (e.g., no residual signals or shuffled-pair comparisons), limiting inference about true inter-brain coupling.	Low Risk: Tasks and VR/RW environments were closely matched. First-person perspective used in VR. Interaction stages were standardized across participants.	Low Risk: Behavioral, neural, and subjective outcomes were thoroughly reported across phases. Brain-behavior correlations included.	Some Concerns: Interpretation of synchrony as social lacks critical control analyses (e.g., shuffled pairs, residual signal methods), increasing the likelihood that observed IBC reflects parallel processing rather than genuine interpersonal coupling.	Some Concerns

## 4 Discussion

### 4.1 The environment’s influence on IBC

#### 4.1.1 Auditory stimuli

The body of research reviewed underscores the complex interplay between external conditions and neural dynamics during social interactions. Music, background noise, interpersonal distance and VR stand out not merely as passive components, but as dynamic orchestraters of neural synchrony among individuals.

The importance of auditory stimuli within an environment was highlighted by the significant impact of music and varying levels (i.e., volumes) of background noise on neural coupling (Khalil et al., 2022; Hu et al., 2022; Chabin et al., 2022; Li et al., 2021, 2023). Music emerged as a mediator of coordination and emotional exchange, facilitating the transmission of emotions and synchronizing neural rhythms among individuals (Khalil et al., 2022; Hu et al., 2022; Chabin et al., 2022). This synchronization extends beyond immediate interactions but results in persistent increases in IBC post-engagement (Khalil et al., 2022). This finding opens new perspectives on the neuroplastic effects of music and its role in maintaining connections between individuals beyond the immediate context of engagement. In addition, the investigation of background noise sheds light on the sophisticated neural networks required for speech processing in noisy conditions, demonstrating that neural coupling intensifies, particularly in the sensorimotor and auditory regions, as background noise level increases (Li et al., 2021, 2023). This neural adaptation facilitates clearer communication by enhancing the brain’s ability to differentiate between meaningful speech and noise. While the focus of these studies was predominantly on the effects of white noise on speech processing, previous research has shown that meaningless noise and meaningful speech interfere with speech comprehension differently (Oswald et al., 2000; Wong et al., 2012). Therefore, future studies comparing the influence of different noise types on neural coupling and speech processing may enhance our understanding of how the brain navigates the complex soundscape of our everyday environments.

#### 4.1.2 Physical proximity

Another notable discovery is the crucial role of physical proximity in IBC (Chabin et al., 2022). Unraveling the precise factors that contribute to heightened IBC when individuals are physically closer is imperative. This prompts inquiries into whether this is due to visual cues, such as seeing someone being closer, or other sensory inputs such as being able to better hear/smell the other person. Moreover, exploring the notion of optimal distances between individuals for maximizing IBC may offer valuable insights into designing environments conducive to promoting teamwork and collaboration.

#### 4.1.3 Virtual reality

Building upon the exploration of natural environments, artificial settings were also relevant within the scope of this review, in particular, VR. The results from Gumilar et al. (2021) suggest that while VR environments can facilitate IBC, the magnitude and distribution of this synchrony may differ from that observed in RW settings. This finding aligns with previous work comparing video-mediated interactions (e.g., Zoom) to in-person meetings, where the neural synchrony observed is generally weaker in virtual contexts (Zhao et al., 2023). This difference could be attributed to the more constrained and less immersive nature of virtual interactions, which may lack the spontaneous and dynamic elements of real-life exchanges such as genuine eye contact and nuanced facial expressions.

However, VR offers distinct advantages that go beyond merely replicating RW scenarios. Pan et al. (2023) demonstrated that VR not only facilitates collaboration but also enhances cognitive synchronization, particularly in areas like the PFC during idea selection. This suggests that VR may enhance decision-making and creative problem-solving by reducing the social pressures often present in face-to-face interactions, allowing participants to feel more secure and willing to share ideas. Despite its limitations in providing tactile and sensory feedback, VR’s flexibility and less restrictive environment fosters originality and satisfaction, particularly during the later stages of collaboration. The fluid exchange of ideas and the ability to shift perspectives within VR also stimulates creative thinking, making it a valuable tool for both research and practical applications.

In contrast, RW environments appear to better support implicit communication through non-verbal social cues like facial expressions and gestures. Higher IBC in the STG observed in RW participants suggests that real-life interactions foster deeper connections without the need for explicit verbal communication. Meanwhile, in VR, increased IBC in the SMG indicates that participants may struggle with interpreting spatial and tactile information due to the limitations of virtual technology. While VR can simulate these elements, it often lacks the realism necessary for precise tactile sensations and spatial awareness, posing challenges for replicating the subtleties of RW communication.

Despite these challenges, VR simulations offer a unique vantage point to investigate how varying degrees of environmental realism affect neural coupling during interactions. Additionally, the versatility of VR in providing diverse visual perspectives, including first-person and third-person views, opens avenues for investigating how these visual shifts influence IBC (Barde et al., 2020). While the literature reviewed does not extensively cover VR, its inclusion points to the growing interest in understanding how technologically mediated environments compare with physical reality in influencing social connection. This inquiry becomes increasingly relevant as our social interactions evolve toward online platforms such as Zoom and the Metaverse and as low-cost VR headsets become increasingly available. Insights from such studies can inform the development of design strategies aimed at promoting IBC in virtual environments, thereby enhancing interaction among users.

#### 4.1.4 Mechanistic integration

The various environmental factors examined in this review appear to influence IBC by engaging both distinct and overlapping brain regions. While the limited number and heterogeneity of studies preclude definitive conclusions, several cross-cutting mechanisms emerge that may help explain how environmental contexts shape neural synchrony. Here, we outline tentative mechanistic themes to support future hypothesis development in this emerging field.

Across studies, music was associated with increased IBC, particularly in delta-band frequencies and in frontal and sensorimotor regions (Hu et al., 2022; Khalil et al., 2022; Chabin et al., 2022). These effects are consistent with rhythmic entrainment, whereby shared auditory rhythms synchronize neural oscillations (Nozaradan et al., 2011). Hu et al. demonstrated that exposure to a musical meter enhanced IBC in the l-MFC, with stronger meters producing greater synchrony, highlighting the role of rhythmic salience. Khalil et al. (2022) found delta-band IBC increased following musical interaction and scaled with interaction duration. This pattern aligns with entrainment-based accounts of social coordination, in which rhythmic stimuli facilitate predictive timing and joint motor planning (Zhang et al., 2024). Future studies could test how altering the tempo or regularity of auditory rhythms modulates the strength and spatial distribution of IBC.

When bottom-up sensory information is degraded or unreliable, the brain may shift toward top-down predictive mechanisms to sustain social understanding. This compensatory adaptation is evident in both background noise and VR studies. Under the most challenging noise condition (−9 dB SNR), stronger coupling in the l-IFG) was associated with better comprehension, whereas this relationship was absent or weak at moderate noise levels (−6 dB) (Li et al., 2021, 2023). Moreover, coupling in classical auditory regions (e.g., MTG, AG) did not predict comprehension performance at any noise level. These findings suggest the brain may engage sensorimotor compensatory mechanisms specifically when auditory input is severely degraded. These findings align with predictive coding accounts of language, in which higher-level brain areas generate top-down predictions to maintain understanding when sensory precision drops (de-Wit et al., 2010). Similarly, VR-based studies revealed reduced IBC in sensorimotor areas, likely due to the limited tactile and visual feedback available in virtual settings. There is instead greater engagement of prefrontal, temporoparietal, and attentional regions, suggesting a shift toward executive and mentalizing systems to support coordination. Building on these findings, future work could examine whether artificially increasing sensory uncertainty (e.g., introducing temporal lag in VR feedback or speech masking with semantically meaningful noise) leads to measurable increases in IBC specifically within top-down control regions.

Finally, Gumilar et al. (2021) demonstrated that first-person perspectives, compared to third-person views, enhanced IBC in social-cognitive and attentional networks. These findings suggest aligning spatial perspective can help to overcome some of VR’s sensory limitations and support social connection. A similar principle may apply in physical environments: Chabin et al. (2022) showed that closer physical proximity during shared music experiences enhanced IBC in frontal and temporoparietal regions. This reduced interpersonal distance likely enhances multisensory social cues, such as facial expressions, gestures, or breathing, thereby facilitating neural alignment between individuals.

### 4.2 Methodological challenges and opportunities

#### 4.2.1 Variability in imaging approaches, analyses and reporting standards

In the compilation of papers reviewed, there is a notable equilibrium between the employment of fNIRS and EEG. Nonetheless, fNIRS has emerged as the predominant choice in the broader landscape of hyperscanning research (Hakim et al., 2023). This preference is likely due to its inherent wearability and resistance to motion artifacts, making it ideal for studying dynamic social interactions, especially in naturalistic settings (Pinti et al., 2018). It is noteworthy that the sole paper reviewed conducted in a natural environment, employed EEG (Chabin et al., 2022), however, as participants remained seated during the simulated concert, concerns regarding movement artifacts were minimal. Moving forward, there is an opportunity for researchers to employ fNIRS in naturalistic environments, for example investigating the impact of green spaces versus indoor spaces on IBC. Nonetheless, while offering clear advantages in ecological validity, such experiments also introduce a spectrum of challenges and considerations, as highlighted by Pinti et al. (2018). Developing advanced data analysis techniques, such as wavelet-based filtering and tPCA, to correct for motion artifacts and integrating fNIRS with other physiological monitoring tools will be essential to ensure the accuracy and reliability of data collected in naturalistic settings (Molavi and Dumont, 2012; Yücel et al., 2014).

Alongside the varied choice of imaging modalities, there exists a wide range of signals of interest and analysis algorithms utilized to quantify IBC. The absence of established norms and practices can be attributed to the lack of a unified theoretical framework of IBC (Holroyd, 2022). Without widely accepted theories and models of IBC, studies lack solid mechanistic foundations, raising the risk of disseminating results that may be misinterpreted or misleading.

Additionally, a key methodological insight from this review is the substantial variability in how environmental manipulations and IBC findings are reported across studies. This heterogeneity poses a major barrier to cumulative science, making it difficult to compare results or conduct quantitative syntheses. We strongly recommend future research adopts standardized reporting practices, including clear descriptions of environmental variables, consistent IBC metrics, and common frameworks for brain region labeling (e.g., using MNI coordinates or standard atlases). Such harmonization will greatly enhance reproducibility, transparency, and the feasibility of future meta-analytical work in this emerging field.

#### 4.2.2 Disentangling common input and task confounds

Another key methodological challenge across the reviewed studies involves disentangling genuine IBC from common input confounds. In many paradigms, such as shared musical listening or exposure to the same virtual scene, both participants receive identical exogenous stimuli. These shared inputs can evoke parallel, stimulus-locked neural responses, potentially masquerading as IBC. This issue is particularly problematic when studies report zero-lag coherence without implementing control analyses that differentiate true dyadic coupling from coincident entrainment. To address this, future research must incorporate essential control analyses, such as shuffled-partner comparisons, cross-brain Granger causality, lag-based coherence estimates, and asymmetric stimulus designs (e.g., using different stimuli across participants). These tools help establish whether observed IBC genuinely reflects dynamic interpersonal processes rather than shared sensory alignment. For example, comparing dyads exposed to identical versus divergent musical tracks, or using yoked-replay paradigms where one partner’s behavior is simulated, can help isolate socially driven IBC. The asymmetric design used by Li et al. (2021, 2023) offers a partial solution: only listeners were exposed to background noise, while speakers narrated under normal conditions. This setup avoids full common-input confounds and isolates listener-driven neural adaptation mechanisms. However, it departs from ecologically realistic in-person conditions in which both partners are typically exposed to environmental noise, possibly limiting the generalizability of the results.

Beyond common input, a related but distinct challenge is differentiating IBC arising from interactive processes versus that driven solely by task demands. This is especially critical in motor-heavy paradigms. For example, Khalil et al. (2022) addressed this by measuring brain activity only before and after the tapping task, allowing them to attribute post-task IBC changes to the interaction itself, rather than to concurrent movement/motor synchrony. This was not taken into consideration in other studies reviewed whereby the task involved motor activity, potentially limiting the reliability of their results (Gumilar et al., 2021; Hu et al., 2022).

#### 4.2.3 The “synchrony = social communication” fallacy

A recurring issue in IBC research is the overinterpretation of neural synchrony as definitive evidence of social interaction. While many studies equate significant IBC with meaningful interpersonal communication, synchrony can arise through several alternative, non-social mechanisms. For example, as previously noted, participants performing identical or highly structured tasks may exhibit similar neural responses due to shared cognitive demands rather than true interaction. Moreover, even in the absence of identical stimuli, participants might adopt similar cognitive strategies, such as counting, focusing attention, or rehearsing content, which can lead to convergent neural patterns that reflect parallel engagement. Additionally, shared autonomic rhythms, such as synchronized breathing or heart rate, can influence slow cortical dynamics and bleed into neuroimaging signals, especially in fNIRS and EEG. These physiological rhythms, if not accounted for, can mimic neural coupling. Non-neural artifacts such as volume conduction in EEG or hemodynamic signal spread in fNIRS also pose risks, particularly when participants are in close physical proximity, as overlapping signals can create the illusion of coupling even when neural activity is not coordinated.

Several methodological strategies can help guard against the overinterpretation of synchrony as social interaction. For example, Zhao et al. (2023) employed a residual signal approach similar to Psychophysiological Interaction (PPI) analysis (Friston et al., 1997), removing task-evoked activity before assessing coupling. They also implemented shuffled-dyad comparisons to confirm that observed coherence was specific to real partner pairs. Such rigorous controls are notably underused in the reviewed literature and should be standard practice in future hyperscanning studies aiming to isolate genuine interaction-specific effects.

Beyond analytical techniques, addressing this interpretive challenge will require collaborative replication efforts and a clear, operational definition of what constitutes IBC. Furthermore, a comprehensive understanding of IBC will demand multi-modal data integration (de Hamilton, 2021). The significance of this was underscored by Guglielmini et al. (2022) and Cañigueral et al. (2021), who demonstrated that IBC often co-varies with changes in systemic physiology and observable behavior, underscoring the need to integrate neural data with other biosignals. The reviewed studies would be strengthened by the inclusion of eye-tracking to capture joint attention, video recordings of motion and facial expressions, and physiological measures such as heart rate or skin conductance to detect shared autonomic states. Only one paper reviewed explicitly mentions measuring physiology (Chabin et al., 2022). Integrating systemic physiology monitoring, particularly into fNIRS studies, is essential to address potential confounding signals from both neuronal and non-neuronal changes. In a recent publication, Scholkmann et al. (2022) introduced Systemic Physiology Augmented fNIRS (SPA-fNIRS) which represents a pivotal method that enables the simultaneous measurement and analysis of fNIRS neuroimaging data alongside systemic physiology data. This approach enhances the interpretation of brain functional activation by providing a more comprehensive understanding of underlying physiological processes.

### 4.3 Implications

The limited number of papers published highlights the relatively early stage of this field, likely due to challenges associated with experimental protocols, the only recent feasibility of such studies, and funding limitations. Nevertheless, this review underscores the critical importance of considering environmental factors in the study of social interactions and the need for broader exploration.

The findings presented open the door to a new genre of experiments aimed at advancing our understanding of brain-environment interactions and their role in shaping social behavior. Environmental features such as lighting, spatial layout, green spaces and climate may all impact IBC and, by extension, interpersonal dynamics. These elements, both individually and collectively, warrant investigation in both controlled experimental designs and ecologically valid settings. Additionally, investigating coherence across physiological and behavioral markers, such as synchrony in pupil diameter, heart rate alignment, or breathing rate, could complement neural findings and provide valuable insights into shared states and their relationship to social cohesion.

Beyond foundational science, this research holds translational potential across clinical and applied settings. For example, examining how environmental modifications affect social engagement in populations with social-skill deficits, such as individuals with ASD or schizophrenia, may inform low-cost, non-invasive strategies to enhance social engagement. Interventions could include using music during interactions, adjusting interpersonal distance, or reducing sensory load. Additionally, VR platforms, through shared visual perspectives or structured joint tasks, may offer promising tools for social skills training in clinical or developmental populations. In mental health, educational, and workplace contexts, insights from this research can inform practical design choices, including spatial layout, acoustic conditions, and communication tools, to improve interpersonal connection and coordination across settings.

This line of research aligns with a forward-looking vision of social neuroscience that emphasizes studying brain function in RW contexts. By moving beyond traditional, controlled laboratory settings and integrating neural, physiological, behavioral, and environmental measures, researchers can better capture the complexity of human experiences. This shift toward a more integrative and holistic approach will not only deepen scientific understanding but may also hold practical implications for designing interventions and environments that promote mental health and positive social interactions in everyday life.
